# From pocket to profile: Scale-free pelvic sex estimation from 3D photogrammetry

**DOI:** 10.1007/s00414-026-03860-8

**Published:** 2026-06-05

**Authors:** Michal Kuchař, Alexander Morávek, Anežka Pilmann Kotěrová, Eugénia Cunha, Tomáš Janoušek

**Affiliations:** 1 Department of Anatomy, Faculty of Medicine in Hradec Králové, Charles University, Šimkova 870, Hradec Králové, 500 03 Czech Republic; 2 Department of Anthropology and Human Genetics, Faculty of Science, Charles University, Viničná 7, Prague, 128 44 Czech Republic; 3 Department of Life Sciences, Centre for Functional Ecology (CFE), Laboratory of Forensic Anthropology, University of Coimbra, Calçada Martim de Freitas, Coimbra, 3000-456 Portugal; 4 Instituto Nacional de Medicina Legal e Ciências Forenses, IP, Lisabon, 1169-201 Portugal

**Keywords:** Pelvic sex estimation, Os coxae, Photogrammetry, Scale-free analysis, Forensic anthropology

## Abstract

**Background:**

Biological sex estimation is a cornerstone of the forensic biological profile, and the adult pelvis remains the most informative skeletal region due to pronounced sexual dimorphism. At the same time, three-dimensional documentation of skeletal evidence is rapidly expanding beyond computed tomography and laboratory scanners toward inexpensive, accessible approaches such as photogrammetry—with mobile phone cameras. While the reliability of photogrammetry for osteological documentation has been supported, most established pelvic sexing approaches still assume measurements in absolute units or rely on expert scoring of morphological traits.

**Methods:**

We propose and validate a scale-free workflow for sex estimation from unscaled 3D pelvic models. The final approach is based on within-specimen log-ratios of seven collected pelvic measurements rather than on absolute units. Model development used a pooled reference dataset of 2,321 known-sex os coxae from documented dry-bone and CT-derived sources, and external implementation testing targeted unscaled photogrammetric pelvic models acquired under realistic conditions (*n* = 103).

**Results:**

In the Coimbra photogrammetric subset, the final scale-free model achieved 97.1% binary accuracy, 97.1% balanced accuracy, and an area under curve of 0.997. Under conservative strict thresholds, 90.3% of cases received a definitive classification, all of which were correct, while the remaining 9.7% were retained as indeterminate.

**Conclusions:**

Scale-free pelvic sex estimation supports the forensic use of unscaled photogrammetric 3D bone models in situations where reliable external calibration is unavailable. The method does not replace standard calibrated osteometry, but it provides a practical and defensible alternative for low-cost digital documentation, remote analysis, and triage-oriented forensic workflows.

**Supplementary Information:**

The online version contains supplementary material available at https://doi.org/10.1007/s00414-026-03860-8.

## Introduction

Sex estimation from human skeletal remains is a foundational step in constructing the biological profile in both forensic anthropology and bioarchaeology. Among adult skeletal elements, the os coxae is widely regarded as the most informative region for this purpose because pelvic morphology exhibits pronounced sexual dimorphism associated with obstetric and functional constraints. Consequently, the pelvis continues to occupy a central role in methodological research on skeletal sex assessment and in practical casework applications.

Classical approaches to pelvic sex estimation include both morphological and metric methods. Morphological methods are based on the visual assessment of sexually dimorphic features of the os coxae, among which the pubic traits described by Phenice [1] remain particularly influential. Metric methods, in contrast, employ linear measurements within discriminant or probabilistic frameworks to provide a more standardized basis for sex estimation. Among them, the Diagnose Sexuelle Probabiliste (DSP) framework and its later development DSP2 are particularly important because they explicitly model population variability and provide probabilistic output rather than a strictly dichotomous assignment [2, 3]. Their performance has been validated in multiple populations and study settings, supporting the broader value of pelvic metric methods when the relevant landmarks can be measured reliably [4–7].

Over the past two decades, forensic anthropology and osteology have undergone a substantial virtual turn. Postmortem computed tomography, surface scanning, and photogrammetry now make it possible to document skeletal material in digital form, preserve fragile specimens, share data more efficiently, and perform measurements directly in virtual environments [8–10]. This development has stimulated virtual anthropology as a mature methodological field and has expanded the practical use of three-dimensional models in both research and forensic documentation [9]. Furthermore, recent work has shown that virtual workflows can support landmarking, automated or semi-automated variable extraction, and sex-estimation procedures derived from established osteometric frameworks [11–14].

At the same time, the increasing use of virtual models raises a practical methodological question: whether current sex-estimation tools are fully adapted to the types of 3D models now being generated in routine practice. This issue is especially relevant for photogrammetric workflows, which are attractive because they are comparatively inexpensive, portable, and feasible in field or collection settings, but may not always provide reliable metric scaling [15, 16].

A major obstacle for the broader forensic use of photogrammetric bone models is therefore that many models are effectively unscaled. When absolute size cannot be trusted, conventional metric approaches become difficult to apply because the biological signal is confounded with reconstruction scale. This problem is particularly important for pelvic sex estimation, where many successful models rely at least partly on absolute dimensions or on methods that assume metrically calibrated input. One possible solution is to shift the analytical emphasis from raw size to within-specimen proportional structure, using scale-normalized variables that preserve shape-related information while reducing dependence on absolute magnitude [17–20].

The present study addresses this gap by developing a practical scale-free approach to pelvic sex estimation for use on unscaled 3D models. Specifically, we evaluate whether sex can be estimated from a reduced set of linear pelvic variables transformed into within-specimen log-ratios, thereby making the method portable across measurement contexts in which reliable scaling is unavailable. In this sense, the study is motivated not only by theoretical interest in size-normalized morphometrics, but also by a pragmatic forensic need: to enable biologically meaningful sex estimation from digitally reconstructed pelvic remains in situations where low-cost image-based 3D capture is feasible, but full metric calibration is not.

## Materials and methods

This study was designed as the development and implementation testing of a probabilistic metric approach to sex estimation from the adult os coxae. The analytical workflow was built on pooled known-sex pelvic data from documented osteological collections, complemented by virtual pelvic material measured in a comparable way.

### Development sample

**Fig. 1:**
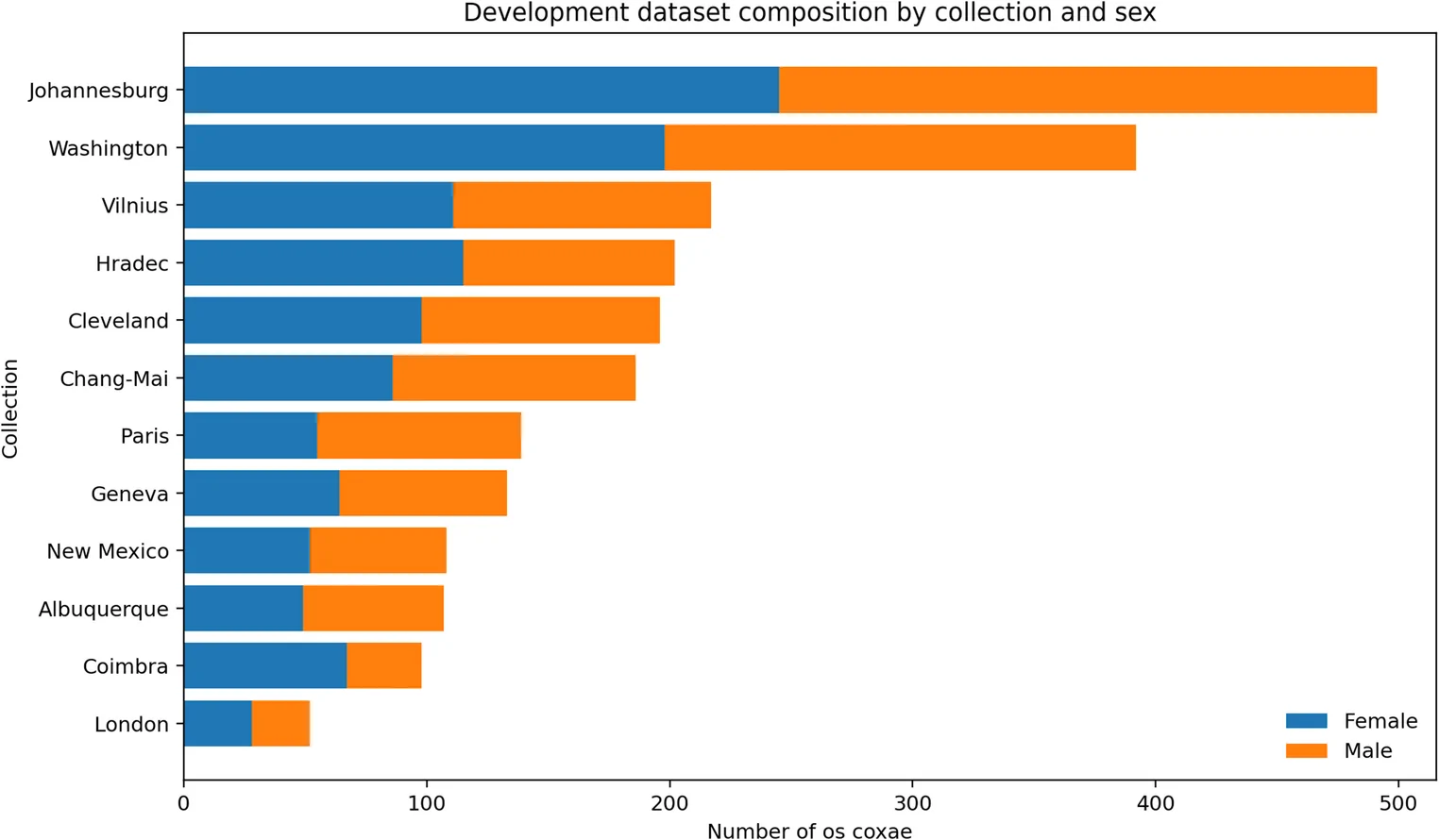
Composition of the development dataset by collection and sex

The development dataset contained 2,321 complete os coxae observations from 12 reference groups after preparation of the pooled file and exclusion of directly matched dry-bone Coimbra cases. Sex distribution was nearly balanced (1,153 males and 1,168 females). The largest contributions came from Johannesburg (*n* = 491), Washington (*n* = 392), Vilnius (*n* = 217), Hradec Králové (*n* = 202), Cleveland (*n* = 196) and Chang-Mai (*n* = 186), whereas London represented the smallest reference subset (*n* = 52). The dry-bone data are publicly available as an appendix to the DSP article [3] and were used with the author’s (J. Brůžek) prior approval. Figure 1 summarizes collection-level sample composition, whereas sex-specific mean values for the retained variables are reported in Table 1.

**Table 1:** Collection-level summary of the development sample (in millimetres) and Coimbra validation subset (unit-less). Values are sex-specific means for the seven retained variables; the Coimbra validation subset is shown separately at the bottom

Collection	Total (*n*)	PUM	SPU	DCOX	ISMM	SCOX	SS	SA
Albuquerque	107	F 72.45 M 72.17	F 25.21 M 30.24	F 198.02 M 221.14	F 99.93 M 115.08	F 153.63 M 160.38	F 70.73 M 77.28	F 78.33 M 79.54
Chang-Mai	186	F 72.34 M 66.78	F 25.19 M 29.38	F 190.57 M 203.25	F 96.53 M 106.31	F 146.50 M 146.85	F 65.76 M 71.43	F 74.55 M 73.05
Cleveland	196	F 74.15 M 70.92	F 25.67 M 30.23	F 201.27 M 217.86	F 102.87 M 114.55	F 155.16 M 159.43	F 69.82 M 74.92	F 76.45 M 74.44
Coimbra (development reference subset)	98	F 70.58 M 68.77	F 22.73 M 27.84	F 196.99 M 215.00	F 99.04 M 111.23	F 152.84 M 156.97	F 64.04 M 72.81	F 72.49 M 74.06
Geneva	133	F 74.69 M 72.57	F 24.59 M 29.53	F 202.80 M 220.91	F 101.88 M 115.06	F 157.45 M 160.77	F 70.00 M 75.68	F 78.43 M 77.37
Hradec	202	F 77.14 M 74.96	F 24.08 M 31.36	F 206.90 M 225.93	F 106.10 M 119.73	F 159.87 M 165.53	F 71.68 M 80.01	F 75.56 M 79.14
Johannesburg	491	F 69.54 M 67.03	F 23.69 M 28.26	F 191.20 M 205.87	F 98.98 M 108.70	F 145.02 M 149.34	F 65.57 M 70.73	F 71.67 M 71.67
London	52	F 72.59 M 71.72	F 24.30 M 30.29	F 193.54 M 212.38	F 99.93 M 113.19	F 150.11 M 153.67	F 66.92 M 73.75	F 73.93 M 75.92
New Mexico	108	F 74.12 M 74.35	F 24.29 M 30.36	F 210.23 M 229.06	F 104.18 M 117.49	F 148.96 M 160.37	F 72.56 M 80.54	F 76.40 M 80.69
Paris	139	F 73.49 M 72.27	F 24.02 M 30.15	F 201.80 M 219.93	F 102.11 M 114.90	F 155.20 M 162.42	F 67.69 M 75.61	F 75.71 M 75.64
Vilnius	217	F 76.19 M 72.88	F 25.16 M 31.12	F 205.91 M 223.47	F 105.41 M 116.90	F 162.19 M 165.13	F 69.35 M 76.01	F 77.96 M 77.09
Washington	392	F 72.84 M 71.33	F 25.23 M 30.17	F 198.75 M 217.37	F 102.61 M 115.01	F 153.66 M 157.96	F 68.94 M 76.41	F 76.00 M 76.66
*Coimbra (validation subset)*	*103*	*F 1.56* *M 1.51*	*F 0.47* *M 0.60*	*F* 4.24 *M 4.59*	*F 2.13* *M 2.39*	*F 3.28* *M 3.31*	*F 1.42* *M 1.55*	*F 1.55* *M 1.49*

### Validation sample

An independent external validation dataset was derived from the Coimbra Identified Skeletal Collection (CISC) (see the subsection *Photogrammetric Validation* for details). Because some pelvic bones from this collection overlapped with the development dataset, those individuals were excluded. Following this step, 98 Coimbra dry-bone cases remained in the pooled reference sample for model development, and 103 newly generated models were retained for external validation. Side representation was balanced (50 right and 53 left os coxae).

### Pelvic variables and measurement definitions

The methodological framework was restricted to linear pelvic dimensions that can be routinely collected from the os coxae and applied consistently across physical and virtual materials. Although a broader variable set was considered during model development, the final workflow retained seven measurements: Acetabulo-symphyseal pubic length (PUM), Cotylo-pubic breadth (SPU), Maximum pelvic height (DCOX), Post-acetabular ischium length (ISMM), Iliac breadth (SCOX), spino-sciatic length (SS), and spino-auricular length (SA). These variables follow the DSP osteometric tradition [2], although their original definitions predate DSP itself. PUM, DCOX, and SCOX derive from Bräuer [21], SPU, SS, and SA from Gaillard [22], and ISMM from Schulter-Ellis et al. [23]. Although IIMT, depth/height of the greater sciatic notch, showed strong predictive performance, it was excluded from the final model because of its lower transferability and the added complexity it would have introduced into the workflow [13]. Only specimens with all seven retained measurements available were included in classification. Because measurement procedures on 3D models may differ from those applied to dry bones, a detailed measurement guide is provided in Online Resource 1.

### Scale normalization and model development

Because the intended workflow needed to remain robust across heterogeneous collections and measurement contexts, the final predictors were expressed as within-specimen log-ratios rather than raw absolute dimensions. Following the Mosimann framework for size normalization [17], measurement SS served as the reference variable, and all retained dimensions were entered as $\:\mathrm{ln}\left(\frac{X}{SS}\right)$. SS was selected a priori as the denominator because it was retained in the final reduced variable set, showed high measurement repeatability in the photogrammetric observer-error study, and represents a stable pelvic dimension that can be consistently identified on unscaled 3D models. The choice was therefore practical and reproducibility-oriented rather than intended to assign special biological primacy to SS. This strategy reduces sensitivity to reconstruction scale while preserving biologically meaningful proportional relationships among pelvic dimensions. The same denominator was applied consistently to both the development and validation data.

Sex classification was modelled using binary logistic regression. To limit distortion arising from marked imbalance across collections and within collection-specific sex groups, model fitting employed a weighting scheme that gave more balanced influence on each collection-by-sex stratum than would have been obtained under simple pooling. CT-derived material from Hradec Králové and New Mexico [13, 24], based on template-driven automatic measurements, was assigned a lower weight (0.5) than the conventional dry-bone reference groups. This approach allowed these data to contribute to model development while reducing potential bias associated with automatically generated measurements.

### Photogrammetric validation subset and observer error study

To evaluate the practical applicability of the proposed workflow, a photographic dataset of pelvic bones was created from the Coimbra Identified Skeletal Collection (CISC). Each specimen was documented by standardized multi-view photography using a smartphone camera (Google Pixel 9). For each specimen, approximately 160 photographs were acquired. The workflow was based on standardized close-range photogrammetric procedures for osteological material and on published low-cost and repeatable reconstruction approaches and was implemented here in a smartphone-based acquisition setup [15, 25, 26]. The photographs were subsequently processed in Agisoft Metashape to generate a single three-dimensional surface model for each specimen. Final meshes were simplified to 100,000 triangular faces as a practical compromise between geometric detail and efficient downstream processing and were exported into STL format for subsequent analysis. The final analyzable photogrammetric dataset comprised 103 models, all of which were manually measured for the seven retained variables.

For evaluating the repeatability of DSP-defined distance measurements, a subset of 35 models was randomly selected from the complete dataset for observer error analysis. These models were independently measured by three observers to evaluate interobserver error. The same subset was then remeasured by the same observers after an interval of 14 days to assess intraobserver error.

Observer agreement was evaluated using standard reliability statistics. Intraobserver and interobserver reliability for continuous measurements were quantified using the intraclass correlation coefficient (ICC) [27, 28]. In addition, the relative technical error of measurement (rTEM) and coefficient of reliability (CR) were calculated to express measurement error [29, 30]. These complementary indices were used to characterize both the consistency and the magnitude of observer-related variation.

### Implementation testing and statistical evaluation

The measurements obtained from all analyzable models were entered into the Sex Estimator, an Excel-based tool developed in this study to implement the final classification equations and decision thresholds (see Online Resource 2). The predicted sex for each specimen was then compared with the known biological sex recorded for the identified collection. The performance of the estimator was evaluated by calculating classification accuracy, including the proportion of correctly classified individuals. The workbook includes an editable strict-threshold parameter, so users can retain the default rule or apply stricter settings according to research or casework requirements. We use 0.90/0.10 as a transparent default threshold for the present scale-free photogrammetric implementation, where additional variability may arise from heterogeneous training data, proportional rather than absolute measurements, and 3D reconstruction/measurement conditions. This threshold is not intended to replace the conventional 0.95/0.05 standard; rather, it provides a conservative but practical default, while the accompanying Excel estimator allows users to modify the PP threshold according to their own evidentiary or institutional requirements.

**Fig. 2:**
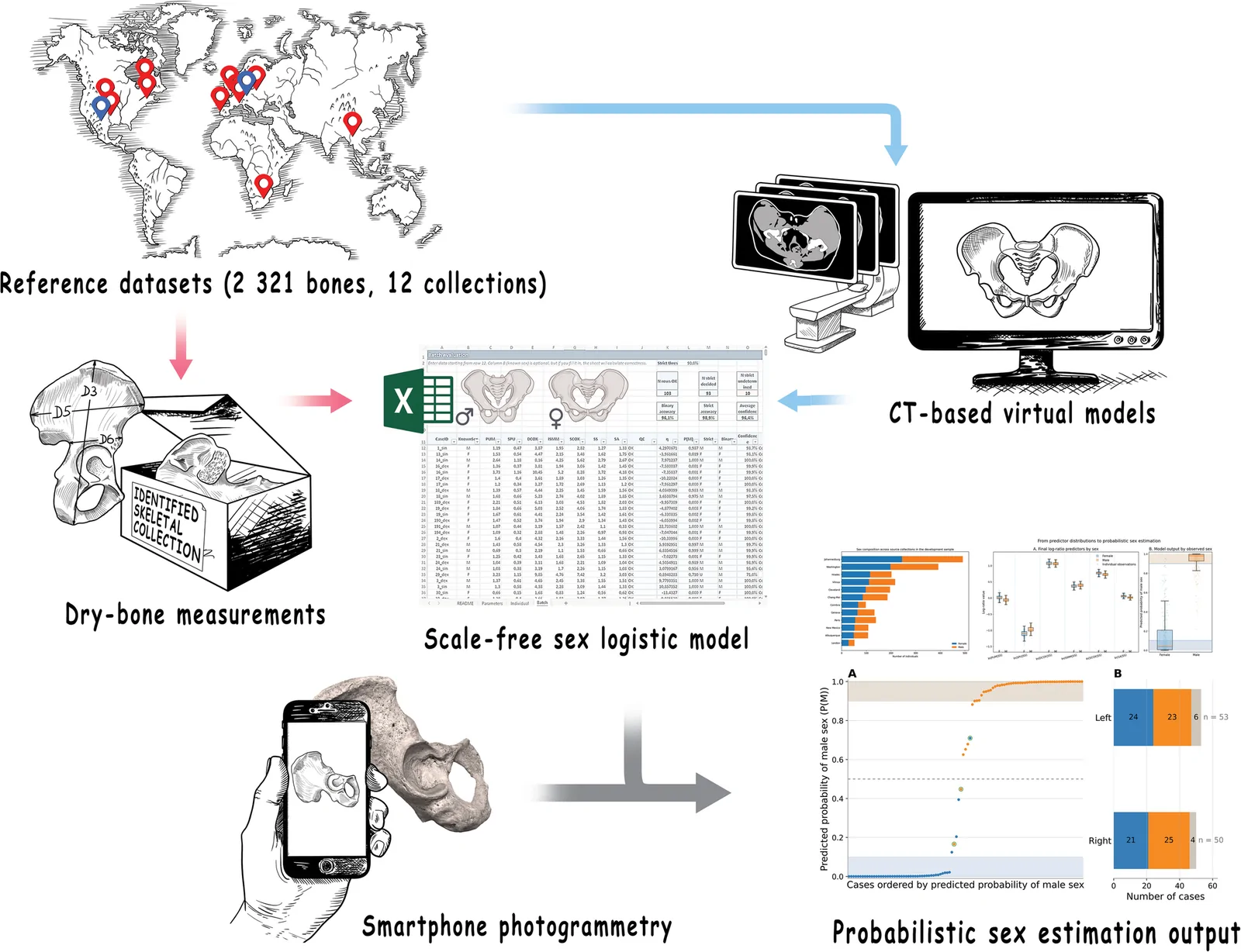
An overview of the full methodological workflow

Because the method can yield male, female, or indeterminate outcomes under strict decision rules, results were assessed in two ways: (1) strict classification performance, in which indeterminate cases were retained as a separate outcome category, and (2) binary classification performance, in which classification was evaluated according to the male/female output used for final sex attribution. The agreement between estimated and known sex was summarized using a confusion matrix. Sensitivity, specificity, balanced accuracy, overall accuracy, and the area under the receiver operating characteristic curve (AUC) were calculated. The AUC was used to assess the overall discriminatory ability of the model across all possible decision thresholds. An overview of the full methodological workflow is presented in Fig. 2.

## Results

### Development and validation sample

All seven retained measurements were complete in the development sample. Pooled mean ± SD values were 71.84 ± 5.46 for PUM, 27.14 ± 3.79 for SPU, 207.35 ± 15.34 for DCOX, 107.48 ± 8.63 for ISMM, 154.95 ± 10.96 for SCOX, 71.55 ± 6.36 for SS, and 75.35 ± 6.46 for SA. Absolute measurements showed the expected pattern of sexual dimorphism, with male pelves presenting larger mean SPU, DCOX, ISMM, SCOX, and SS values, whereas females showed a larger mean PUM; mean SA differed only minimally between the sexes. The photogrammetric data are not scaled in any unit system by default. In our dataset, the mean ± SD values were 1.53 ± 0.58 for PUM, 0.53 ± 0.22 for SPU, 4.43 ± 1.70 for DCOX, 2.26 ± 0.87 for ISMM, 3.29 ± 1.25 for SCOX, 1.49 ± 0.59 for SS, and 1.52 ± 0.60 for SA (Table 1).

**Fig. 3:**
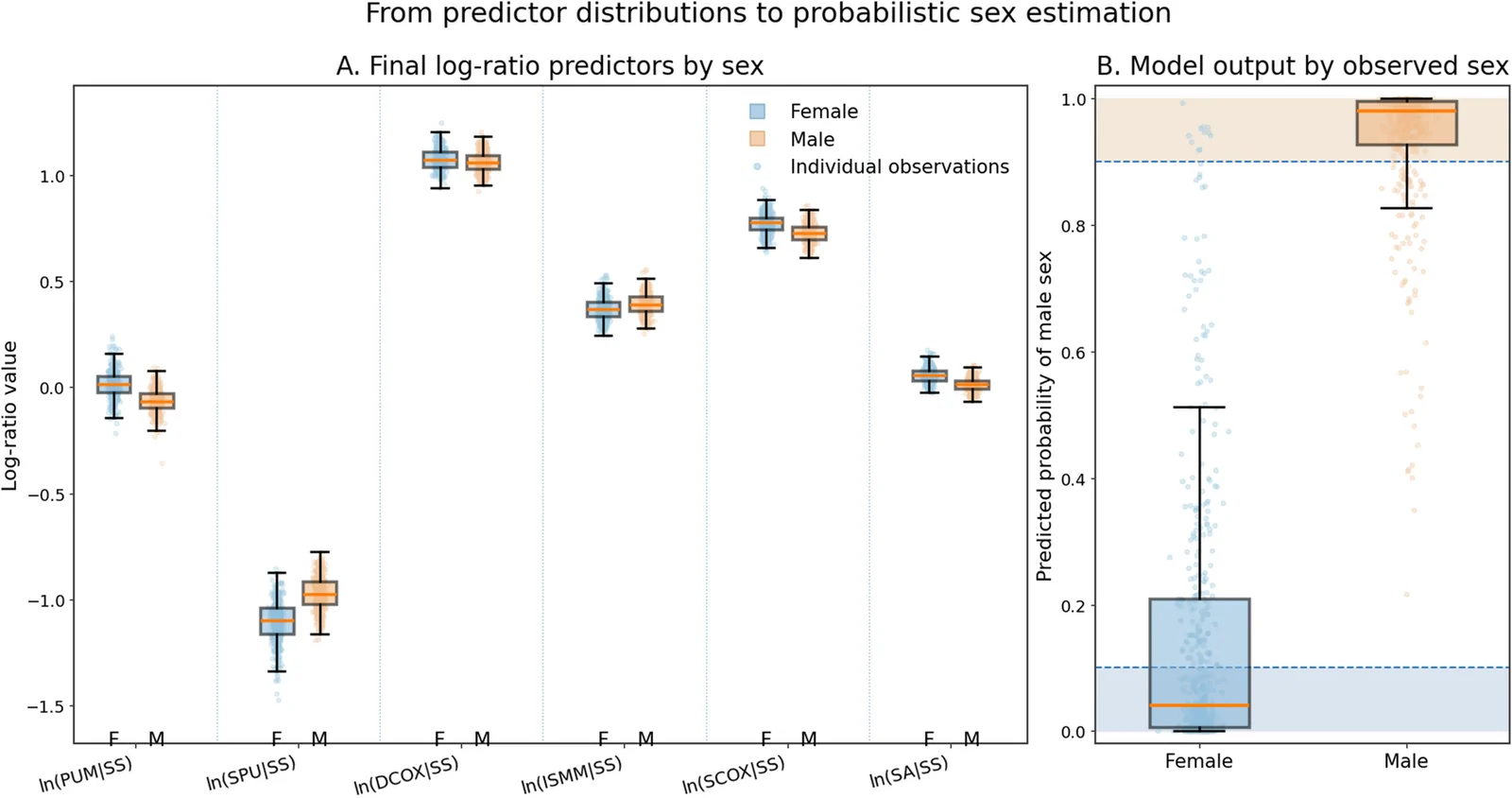
Distribution of retained predictors and resulting model outputs. (A) Sex-specific distributions of the final log-ratio predictors included in the model. (B) Predicted probabilities of male sex by observed sex. The shaded lower and upper bands mark the conservative decision intervals P(M) = 0.00 to 0.10 and P(M) = 0.90 to 1.00

After scale normalization, the clearest sex contrasts were observed for $\:\mathrm{l}\mathrm{n}\left(\frac{PUM}{SS}\right)$, $\:\mathrm{l}\mathrm{n}\left(\frac{SPU}{SS}\right)$, $\:\mathrm{l}\mathrm{n}\left(\frac{SCOX}{SS}\right)$, and $\:\mathrm{l}\mathrm{n}\left(\frac{SA}{SS}\right)$, while $\:\mathrm{l}\mathrm{n}\left(\frac{DCOX}{SS}\right)$ showed only limited univariate separation, indicating that its value was primarily multivariable rather than standalone. The same collection-level summary is provided in Table 1, and Fig. 3 links the observed distributions of the final log-ratio predictors to the probability output of the final model.

### Final classification model

The final linear predictor was defined as

$$\begin{array}{l}{\upeta\:}=-10.19499-46.03852\mathrm{ln}\left(\frac{PUM}{SS}\right)\\+15.29799\mathrm{ln}\left(\frac{SPU}{SS}\right)+29.46989\mathrm{ln}\left(\frac{DCOX}{SS}\right)\\+23.79130\mathrm{ln}\left(\frac{ISMM}{SS}\right)-19.45941\mathrm{ln}\left(\frac{SCOX}{SS}\right)\\-17.58748\mathrm{ln}\left(\frac{SA}{SS}\right)\end{array}$$

The probability of male sex was then calculated as: $\:P\left(\mathrm{Male}\right)=\frac{1}{1+\mathrm{exp}\left(-{\upeta\:}\right)}$.

For routine reporting, specimens were classified as male when P(Male) was at least 0.50 and as female when P(Male) was below 0.50. A conservative strict mode was additionally defined for casework-style interpretation: male for P(Male) ≥ 0.90, female for P(Male) ≤ 0.10, and indeterminate for intermediate probabilities.

### Observer error analysis

Observer agreement in the 35-model repeatability subset was high. Intraobserver ICCs ranged from 0.995 to 0.999, and interobserver ICCs ranged from 0.987 to 1. Intraobserver TEM values were low across all variables, with relative TEMs approximately in the range of 0.52–2.94%, while interobserver rTEMs remained within approximately 0.76–4.9%. The smallest observer-related variation was observed for DCOX and ISMM, whereas SPU and PUM showed the largest—but still acceptable—relative error. The coefficient of reliability shows, in all dimensions, more than 98.8% of variability did not originate as technical error of measurement. A tabulated summary of the observer-error values is provided in Table 2.

**Table 2:** Summary of intraobserver and interobserver measurement error for the seven retained variables

Variable	Intraobserver ICC	Intraobserver TEM (%)	Interobserver ICC	Interobserver TEM (%)	CR
PUM	**0.9962**	**2.69**	**0.997**	**2.53**	**0.9965**
SPU	**0.9981**	**2.02**	**0.987**	**4.90**	**0.9886**
DCOX	**0.9973**	**2.30**	**1.000**	**0.76**	**0.9997**
ISMM	**0.9954**	**2.94**	**1.000**	**0.84**	**0.9996**
SCOX	**0.9999**	**0.52**	**0.999**	**1.30**	**0.9991**
SS	**0.9993**	**1.17**	**0.999**	**1.58**	**0.9987**
SA	**0.9995**	**0.98**	**0.996**	**2.91**	**0.9955**

### Classification performance

Applied to the 103 Coimbra photogrammetric models, the final equation achieved 97.1% binary accuracy (100/103), a balanced accuracy of 97.1%, and an AUC of 0.997. Sensitivity for male classification was 96.3% (52/54), and specificity for female classification was 98.0% (48/49). Binary misclassification was limited to three specimens: two males were classified as female and one female as male under the 0.50 threshold.

**Fig. 4:**
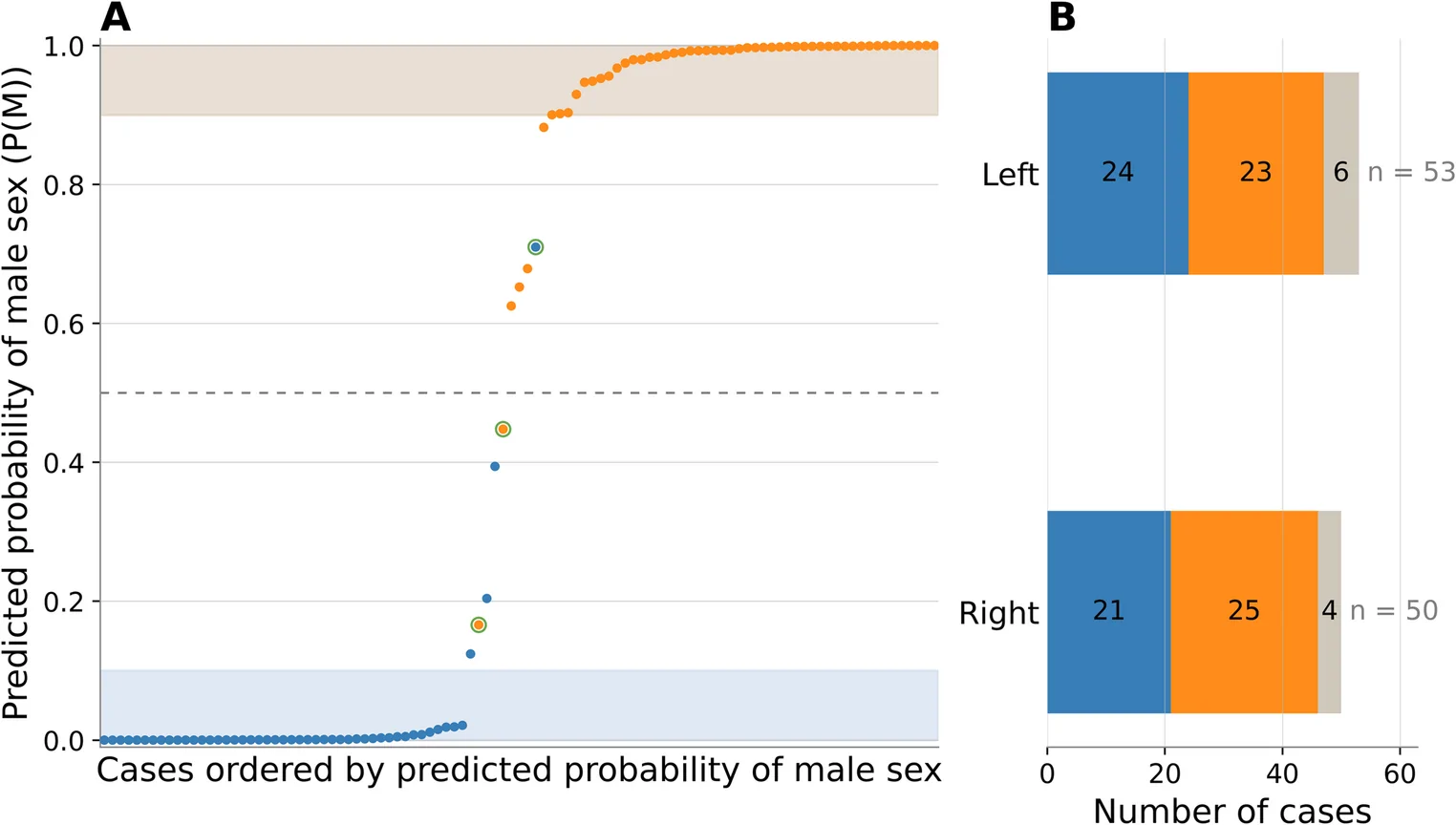
Probability profile of the Coimbra photogrammetric subset and laterality-specific distribution of strict classification outcomes. (A) Ordered predicted probabilities of male sex for all Coimbra cases. Blue points denote females, orange points denote males, and green outlined points indicate binary misclassifications. The dashed line marks the 0.50 decision threshold; shaded bands indicate the strict decision zones. (B) Laterality-specific summary of strict classification outcomes for left and right os coxae

Under the conservative strict rule, 93 of the 103 Coimbra models (90.3%) received a definitive classification and all of these classifications were correct, yielding 100.0% accuracy among decided cases. The remaining 10 cases (9.7%) fell within the indeterminate interval. All three binary errors were absorbed into the indeterminate class under the strict framework, together with seven additional borderline cases (Fig. 4).

## Discussion

### Unscaled 3D models

Our study suggests that even unscaled 3D models obtained with simple photogrammetric workflows retain practical forensic value, provided that their use is aligned with the type of method being applied. This addresses a genuine gap in virtual anthropology. Most validated pelvic metric workflows were designed for calibrated osteometric data, yet the practical use of photogrammetry in collections work, remote documentation, and forensic case management often produces models for which geometric fidelity is acceptable but absolute scale cannot always be guaranteed. At the same time, such models may still support the assessment of sexually dimorphic non-metric pelvic traits by experienced observers, especially when the morphology of the ventral pubis, ischiopubic ramus, subpubic region, and greater sciatic notch is sufficiently preserved [16, 31–33]. Our contribution should therefore be understood as an extension of use rather than as a replacement for established osteometric or virtual workflows. Metric methods such as DSP and DSP2 remain among the most robust and transparent pelvic approaches in forensic anthropology, particularly when explicit probability statements, reproducible thresholds, and defensible expert reasoning are required [2–4, 34].

### Interpretation of main findings

The formula was not constructed as a black-box classifier, but as a deliberately simplified discriminative tool derived from a broad reference sample and anchored in variables that remain interpretable in anatomical terms. We are aware of technically advanced approaches to pelvic sex determination, including machine-learning models, which can produce excellent results when scaled data, collection-specific training, or high-dimensional representations are available [35, 36]. Our objective, however, was different: not to maximize performance within one collection by exploiting all available information, but to preserve as much of the pelvic sex signal as possible under deliberately restricted conditions in which absolute scale is unavailable or uncertain. In that sense, the equation was designed as a pragmatic and transferable model rather than as a collection-specific optimum.

In the Coimbra photogrammetric subset, binary classification achieved 97.1% accuracy (100/103), with 97.1% balanced accuracy and an AUC of 0.997, indicating that the equation retained near-complete discriminatory capacity even when applied to unscaled smartphone-derived photogrammetric models obtained under a standardized low-cost workflow rather than conventionally measured dry bone. This pattern suggests that the model is not simply exploiting overall size tendencies but is capturing a stable component of pelvic sexual dimorphism embedded in relative shape proportions. From an anthropological perspective, that is consistent with the long-standing understanding that the os coxae expresses sex not only through absolute dimensions, but also through patterned structural relationships among pelvic regions. From a forensic perspective, the very small number of errors under the binary rule, only three misclassified pelves, comprising two males classified as female and one female classified as male, shows that the method behaves as a true sex-estimation approach rather than a loose sorting device. Given the specific conditions of our scale-free photogrammetric workflow, we adopted 0.90/0.10 as a transparent default threshold, balancing error control with the proportion of indeterminate cases. However, because this cut-off is a practical decision rule rather than a strictly fixed requirement, users may modify it, for example to the 0.95/0.05 threshold commonly applied in DSP-based studies.

Its forensic value becomes even clearer under the stricter reporting framework. When only sufficiently confident classifications were accepted, 93 of 103 pelves (90.3%) were classified and all 93 were correct, yielding 100.0% strict accuracy among decided cases, while the remaining 10 pelves (9.7%) were appropriately left undetermined. Crucially, the strict rule absorbed all three binary misclassifications into the indeterminate zone, along with seven additional borderline pelves, which means that uncertainty was handled conservatively rather than masked by forced assignment.

### Comparison with previous studies

The present results compare favourably with the broader literature on pelvic metric sex estimation. Classical probabilistic approaches such as DSP and DSP2 generally combine high sexing rates with very high accuracy among classified cases under strict posterior-probability thresholds. In the original DSP study, 90.71% of specimens were sexed with 99.63% accuracy when all 10 variables were available [2]. Comparable dry-bone validations have largely confirmed this pattern, including 85.7% sexing with 97.4% accuracy in a Greek DSP2 sample [4], 83.7% sexing with a 0.4% error rate in a contemporary northeastern Brazilian collection [37], and no sex-assignment errors in a South French sample, although indeterminacy varied strongly with the number of available variables [38]. Comparable studies on virtual models show the same overall tendency. In a modern French CT validation, 92.3% of males and 97.2% of females were sexed correctly, with 100% accuracy among assigned cases [39], while a Danish CT validation achieved a 93.9% sexing rate and 100% accuracy when all 10 variables were used [40].

Against that background, the present study occupies a distinct position: rather than pursuing the maximum performance achievable under scaled CT-derived, high-dimensional, or fully automated conditions, it asks how much of the pelvic sex signal can be preserved when the input consists of unscaled smartphone-derived photogrammetric models and the classifier is intentionally kept simple, anatomically interpretable, and externally testable. In practical forensic use, this makes the workflow potentially relevant for partially skeletonized or otherwise access-limited cases in which the os coxae can be documented sufficiently for targeted virtual measurement, even if the resulting model is not fully complete in all anatomical details. This work is not proposed as a replacement for calibrated surface scanning, CT-based virtual osteometry, or automated extraction pipelines. Instead, its contribution lies in showing that even when the absolute scale is unavailable or uncertain, strong sex-estimation performance can still be retained. For forensic anthropology, osteological curation, and field documentation settings, this represents a meaningful extension of methodological options under constrained acquisition conditions.

### Limitations

The study nevertheless has important limitations. First, a heterogeneous known-sex development sample improves breadth, but it may also blur population- or modality-specific structure. Transportability is gained at the possible cost of finer local calibration. Second, photogrammetric performance depends on image quality, coverage, lighting, surface texture, and reconstruction settings, so not all mobile-phone-derived models should be assumed equivalent. Although the present acquisition workflow was tested under standardized indoor conditions, its main requirements are practical rather than laboratory-specific, and comparable documentation may be achievable outside a formal lab setting provided that the relevant pelvic landmarks remain clearly visible. Third, because the method intentionally departs from an absolute scale, it should not be extrapolated to questions that are intrinsically osteometric. Fourth, although the Coimbra photogrammetric implementation test is an important strength, it does not eliminate the need for replication across additional populations, operators, software environments, and preservation states. Strict sexing rates are threshold-dependent, and posterior probabilities should be interpreted as model-based outputs conditional on the present data and workflow, not as universal error guarantees. Finally, simplicity and interpretability were prioritized over maximal algorithmic performance; as a result, the method should be understood as a robust applied compromise rather than as the endpoint of digital pelvic sex estimation research.

## Conclusions

In conclusion, the present study supports an expanded but carefully delimited role for unscaled photogrammetric pelvic models in sex estimation. Such models may be useful for experienced non-metric assessment and, under the conditions tested here, can also support a simple shape-based statistical approach without claiming to replace established metric standards. The main significance of the work lies in showing that accessibility and forensic usefulness need not be mutually exclusive. By bridging expert morphological practice, probabilistic reasoning, and low-cost virtual acquisition, the study adds a pragmatic methodological tier between traditional dry-bone assessment and more computationally intensive digital pipelines. That tier is likely to be especially valuable in forensic anthropology, osteological curation, and field documentation settings where the best possible model is not always available, but a defensible biological-profile inference is still required.

## Supplementary Information

Below is the link to the electronic supplementary material.

Supplementary Material 1

Supplementary Material 2

## Data Availability

All data supporting the findings of this study are available within the paper and its Supplementary Information.

## References

[1] Phenice TW (1969) A newly developed visual method of sexing the os pubis. Am J Phys Anthropol 30:297–301. 10.1002/ajpa.1330300214 5772048

[2] Murail P, Bruzek J, Houët F, Cunha E (2005) DSP: a tool for probabilistic sex diagnosis using worldwide variability in hip-bone measurements. Bull Mem Soc Anthropol Paris 17:167–176. 10.4000/bmsap.1157

[3] Brůžek J, Santos F, Dutailly B et al (2017) Validation and reliability of the sex estimation of the human os coxae using freely available DSP2 software for bioarchaeology and forensic anthropology. Am J Phys Anthropol 164:440–449. 10.1002/ajpa.23282 28714560

[4] Kranioti EF, Šťovíčková L, Karell MA, Brůžek J (2019) Sex estimation of os coxae using DSP2 software: a validation study of a Greek sample. Forensic Sci Int 297:371.e1-371.e6. 10.1016/j.forsciint.2019.02.011 30851999

[5] San-Millán M, Warrier V, Carrera A, Reina F (2025) Application of DSP2 for biological sex estimation in a Spanish sample: analysis based on sex and side. Int J Legal Med 139:847–862. 10.1007/s00414-024-03358-1 PMC11850468 39545950

[6] Chapman T, Tilleux C, Polet C et al (2020) Validating the probabilistic sex diagnosis (DSP) method with a special test case on Pre-Columbian mummies (including the famous Rascar Capac). J Archaeol Sci Rep 30:102250. 10.1016/j.jasrep.2020.102250

[7] Chapman T, Lefevre P, Semal P et al (2014) Sex determination using the Probabilistic Sex Diagnosis (DSP: Diagnose Sexuelle Probabiliste) tool in a virtual environment. Forensic Sci Int 234:189.e1-189.e8. 10.1016/j.forsciint.2013.10.037 24290894

[8] Grabherr S, Cooper C, Ulrich-Bochsler S et al (2009) Estimation of sex and age of virtual skeletons–a feasibility study. Eur Radiol 19:419–429. 10.1007/s00330-008-1155-y 18766348

[9] Weber GW (2015) Virtual Anthropology. Am J Phys Anthropol 156:22–42. 10.1002/ajpa.22658 25418603

[10] Guyomarc’h P, Samsel M, Courtaud P et al (2017) New data on the paleobiology of the Gravettian individual L2A from Cussac cave (Dordogne, France) through a virtual approach. J Archaeol Sci Rep 14:365–373. 10.1016/j.jasrep.2017.06.005

[11] Decker SJ, Davy-Jow SL, Ford JM, Hilbelink DR (2011) Virtual determination of sex: Metric and nonmetric traits of the adult pelvis from 3D computed tomography models: Virtual determination of sex. J Forensic Sci 56:1107–1114. 10.1111/j.1556-4029.2011.01803.x 21595690

[12] Colman KL, De Boer HH, Dobbe JGG et al (2019) Virtual forensic anthropology: The accuracy of osteometric analysis of 3D bone models derived from clinical computed tomography (CT) scans. Forensic Sci Int 304:109963. 10.1016/j.forsciint.2019.109963 31610335

[13] Kuchař M, Pilmann Kotěrová A, Morávek A et al (2024) Automatic variable extraction from 3D coxal bone models for sex estimation using the DSP2 method. Int J Legal Med 138:2647–2658. 10.1007/s00414-024-03301-4 PMC11490455 39102091

[14] Stan E, Muresan C-O, Dumache R et al (2024) Sex estimation from computed tomography of Os Coxae—validation of the Diagnose Sexuelle Probabiliste (DSP) software in the Romanian population. Appl Sci Basel 14:4136. 10.3390/app14104136

[15] Omari R, Hunt C, Coumbaros J, Chapman B (2021) Virtual anthropology? Reliability of three-dimensional photogrammetry as a forensic anthropology measurement and documentation technique. Int J Legal Med 135:939–950. 10.1007/s00414-020-02473-z 33244707

[16] Braun S, Schwendener N, Kanz F et al (2023) What we see is what we touch? Sex estimation on the pelvis in virtual anthropology. Int J Legal Med 137:1839–1852. 10.1007/s00414-023-03034-w PMC10567926 37336820

[17] Mosimann JE, James FC (1979) New Statistical Methods for Allometry with Application to Florida Red-Winged Blackbirds. Evolution 33:444. 10.2307/2407633 28568176

[18] Jungers WL, Falsetti AB, Wall CE (1995) Shape, relative size, and size-adjustments in morphometrics. Am J Phys Anthropol 38:137–161. 10.1002/ajpa.1330380608

[19] Baur H, Leuenberger C (2011) Analysis of Ratios in Multivariate Morphometry. Syst Biol 60:813–825. 10.1093/sysbio/syr061 PMC3193766 21828084

[20] Klingenberg CP (2016) Size, shape, and form: concepts of allometry in geometric morphometrics. Dev Genes Evol 226:113–137. 10.1007/s00427-016-0539-2 PMC4896994 27038023

[21] Bräuer G (1988) 2. Osteometrie. In: Knußmann R (ed) Anthropologie, 4. Aufl. Gustav Fischer, Stuttgart; Jena; New York, pp 160–232

[22] Gaillard J (1960) Détermination sexuelle d’un os coxal fragmentaire. Bull Mém Société Anthropol Paris 1:255–268. 10.3406/bmsap.1960.1145

[23] Schulter-Ellis F, Schmidt D, Hayek L-A, Craig J (1983) Determination of Sex with a Discriminant Analysis of New Pelvic Bone Measurements: Part I. J Forensic Sci 28:169–180. 10.1520/JFS12249J 6680735

[24] Kuchař M, Henyš P, Rejtar P, Hájek P (2021) Shape morphing technique can accurately predict pelvic bone landmarks. Int J Legal Med 135:1617–1626. 10.1007/s00414-021-02501-6 33502550

[25] Lauria G, Sineo L, Ficarra S (2022) A detailed method for creating digital 3D models of human crania: an example of close-range photogrammetry based on the use of Structure-from-Motion (SfM) in virtual anthropology. Archaeol Anthropol Sci 14:42. 10.1007/s12520-022-01502-9

[26] Feddema JC, Chiu LZF (2024) Accuracy and repeatability of 3D photogrammetry to digitally reconstruct bones. Morphologie 108:100793. 10.1016/j.morpho.2024.100793 38964273

[27] Shrout PE, Fleiss JL (1979) Intraclass correlations: uses in assessing rater reliability. Psychol Bull 86:420–428. 10.1037/0033-2909.86.2.420 18839484

[28] Koo TK, Li MY (2016) A Guideline of Selecting and Reporting Intraclass Correlation Coefficients for Reliability Research. J Chiropr Med 15:155–163. 10.1016/j.jcm.2016.02.012 PMC4913118 27330520

[29] Ulijaszek SJ, Kerr DA (1999) Anthropometric measurement error and the assessment of nutritional status. Br J Nutr 82:165–177. 10.1017/S0007114599001348 10655963

[30] Goto R, Mascie-Taylor CGN (2007) Precision of Measurement as a Component of Human Variation. J Physiol Anthropol 26:253–256. 10.2114/jpa2.26.253 17435374

[31] Klales AR, Ousley SD, Vollner JM (2012) A revised method of sexing the human innominate using Phenice’s nonmetric traits and statistical methods. Am J Phys Anthropol 149:104–114. 10.1002/ajpa.22102 22714398

[32] Buikstra JE, Ubelaker DH (1994) Documentation of Sex Differences and Age Changes in Adults. In: Buikstra JE, Ubelaker DH, Aftandilian D (eds) Standards for Data Collection from Human Skeletal Remains. Arkansas Archeological Survey, Fayetteville, Ark., pp 39–46

[33] White TD, Black MT, Folkens PA (2011) Human osteology, 3rd edn. Academic Press

[34] Obertová Z, Stewart A, Cattaneo C (eds) (2020) Statistics and probability in forensic anthropology. Academic Press. 10.1016/C2017-0-03461-4

[35] Cao Y, Ma Y, Yang X et al (2022) Use of deep learning in forensic sex estimation of virtual pelvic models from the Han population. Forensic Sci Res 7:540–549. 10.1080/20961790.2021.2024369 PMC9639534 36353321

[36] d’Oliveira Coelho J, Curate F (2019) CADOES: an interactive machine-learning approach for sex estimation with the pelvis. Forensic Sci Int 302:109873. 10.1016/j.forsciint.2019.109873 31382223

[37] De Almeida SM, De Carvalho MVD, De Lyra Menezes MCT et al (2020) Validation of the DSP2 tool in a contemporary identified skeletal collection from Northeastern Brazil. Adv Anthropol 10:169–180. 10.4236/aa.2020.102010

[38] Quatrehomme G, Radoman I, Nogueira L et al (2017) Sex determination using the DSP (probabilistic sex diagnosis) method on the coxal bone: efficiency of method according to number of available variables. Forensic Sci Int 272:190–193. 10.1016/j.forsciint.2016.10.020 27856048

[39] Mestekova S, Bruzek J, Veleminska J, Chaumoitre K (2015) A Test of the DSP Sexing Method on CT Images from a Modern French Sample. J Forensic Sci 60:1295–1299. 10.1111/1556-4029.12817 26258990

[40] Rodriguez Paz A, Banner J, Villa C (2019) Validity of the probabilistic sex diagnosis method (DSP) on 3D CT-scans from modern Danish population. Rev Médecine Légale 10:43–49. 10.1016/j.medleg.2018.08.002

