# Supplementary material for: From pocket to profile: Scale-free pelvic sex estimation from 3D photogrammetry
Source: Int J Legal Med. 2026 Jun 5;140(6):3983–92. doi: 10.1007/s00414-026-03860-8 (PMC13658309; doi:10.1007/s00414-026-03860-8)
Supplement: Supplementary file 1 [file 414_2026_3860_MOESM1_ESM.pdf]

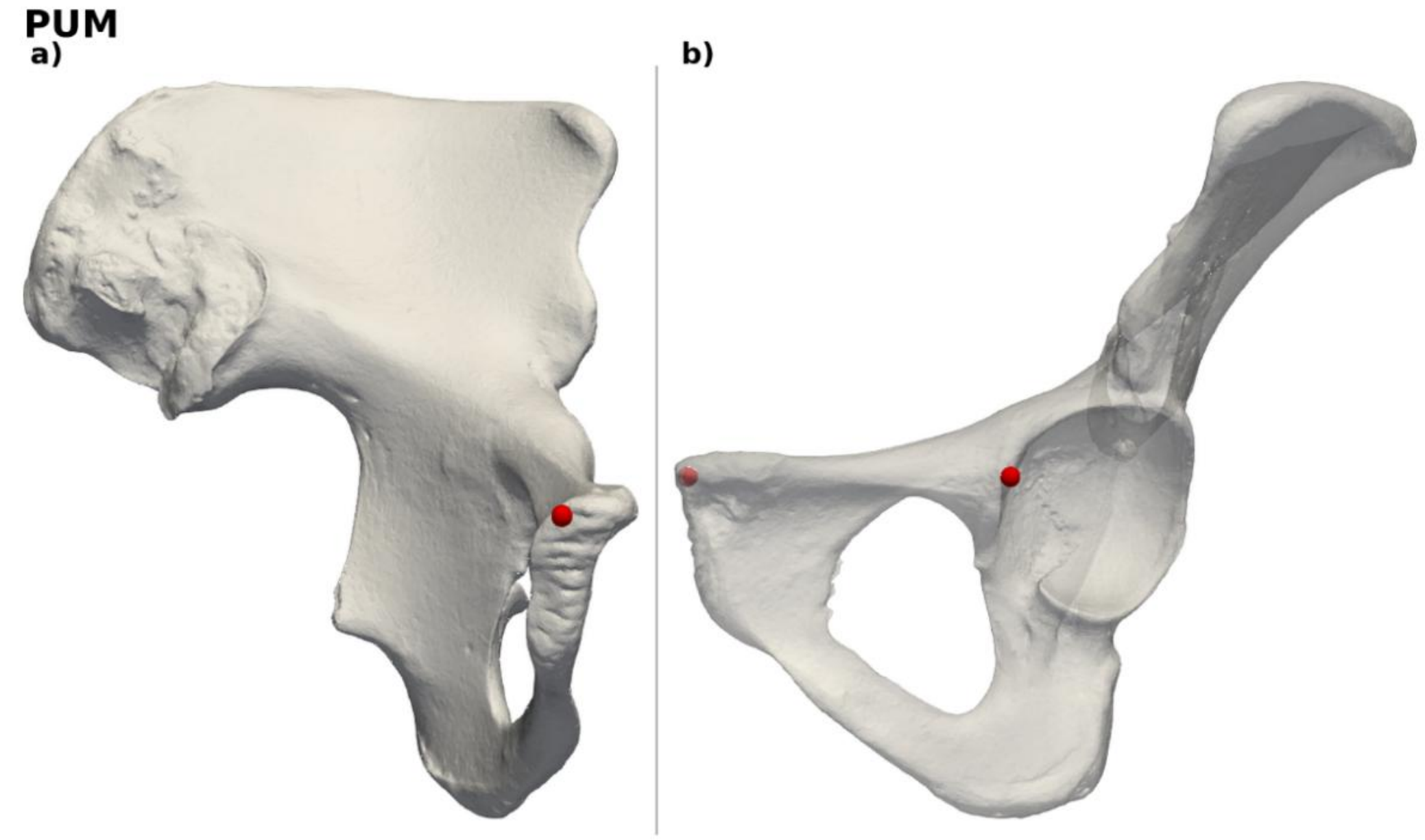

PUM: **(a)** Identify the superomedial point of the pubic symphysis and **(b)** the nearest point on the acetabular rim.

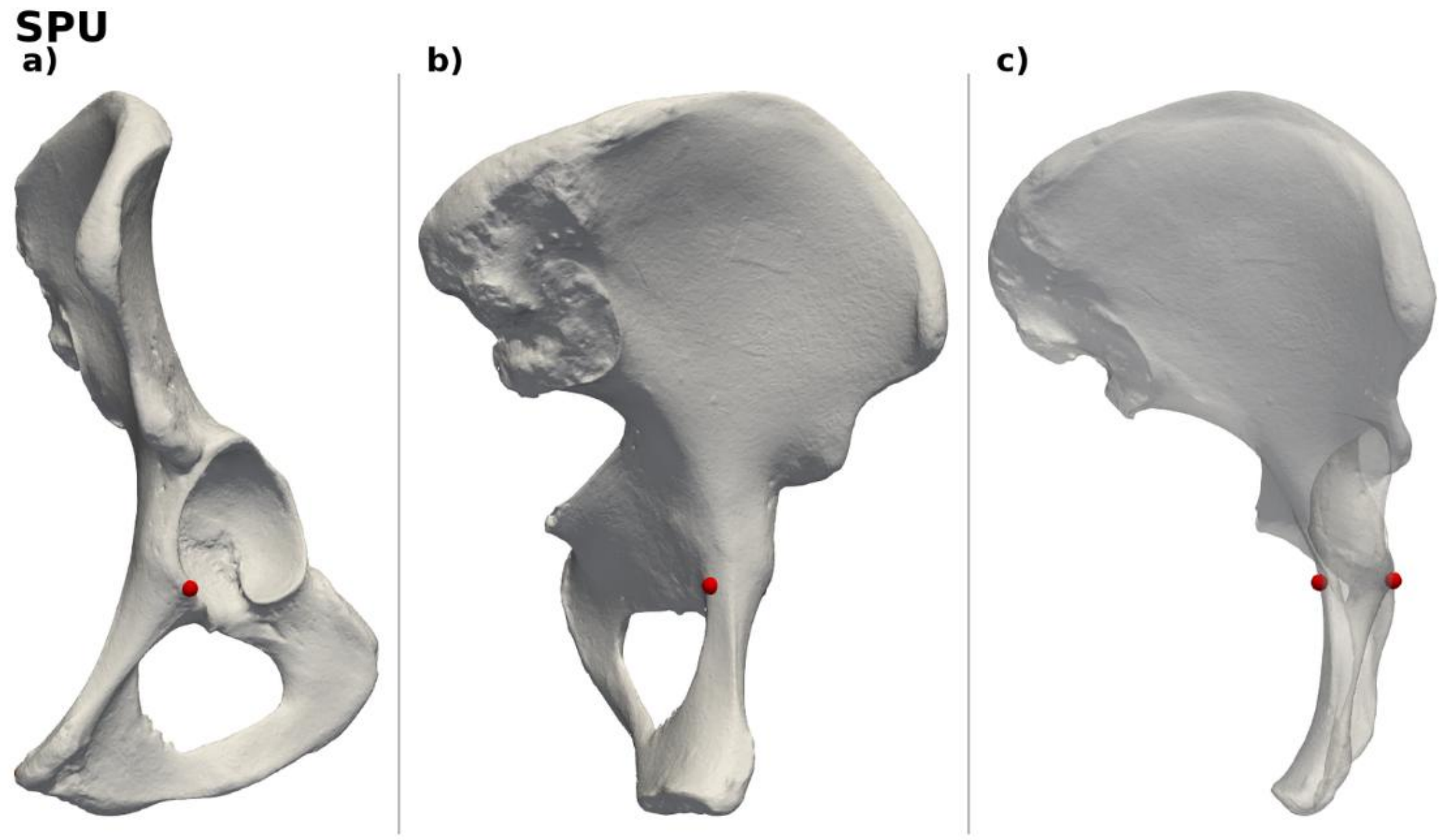

SPU: **(a)** Identify the most lateral acetabular point on the pubic part, **(b)** the medial wall of the pubic margin, and (c) align them perpendicular to the main axis of the pubis.

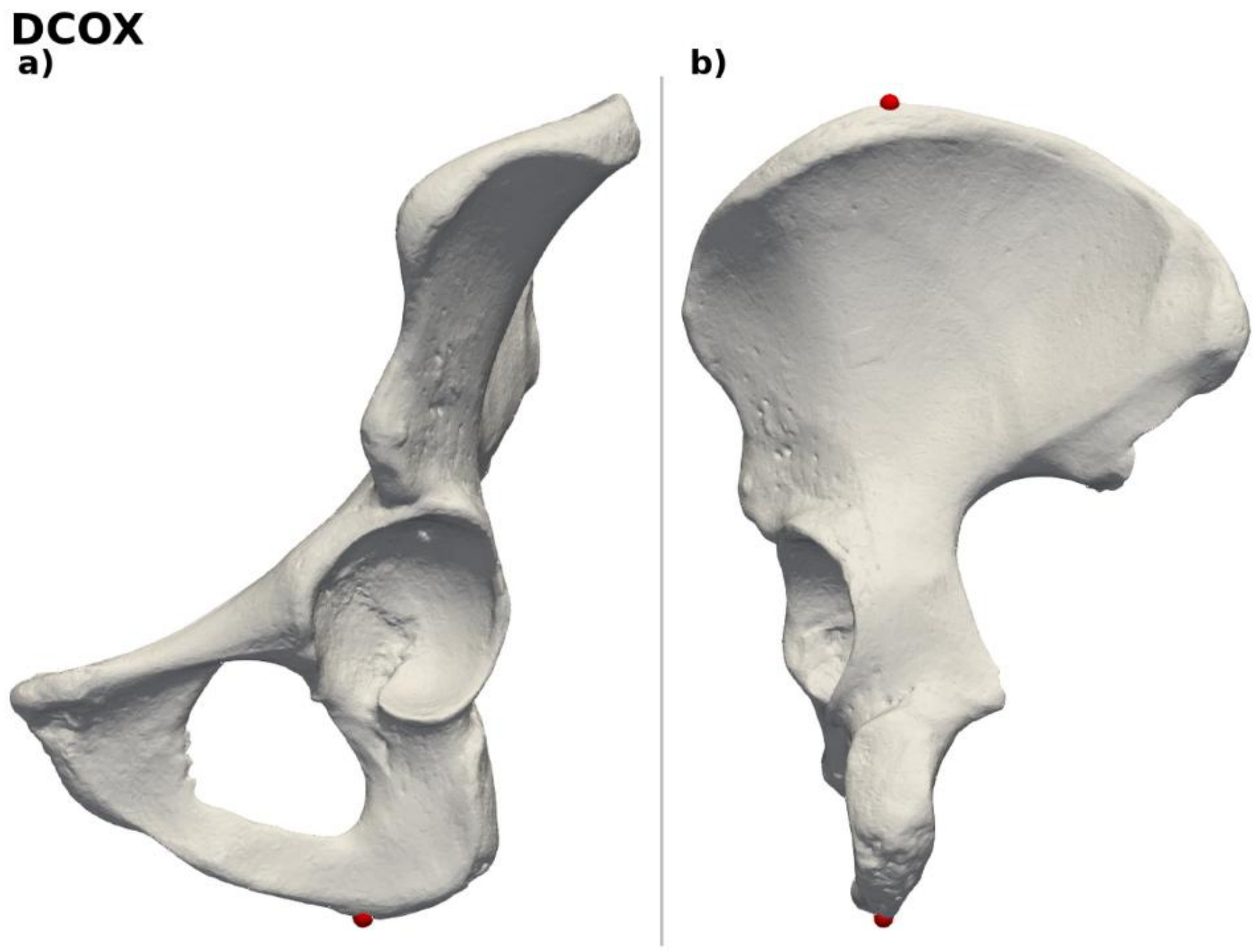

DCOX: (a) Rotate the bone along its main axis to identify the lowest point on the inferior border of the os coxae and **(b)** rotate it to identify the highest point on the iliac crest.

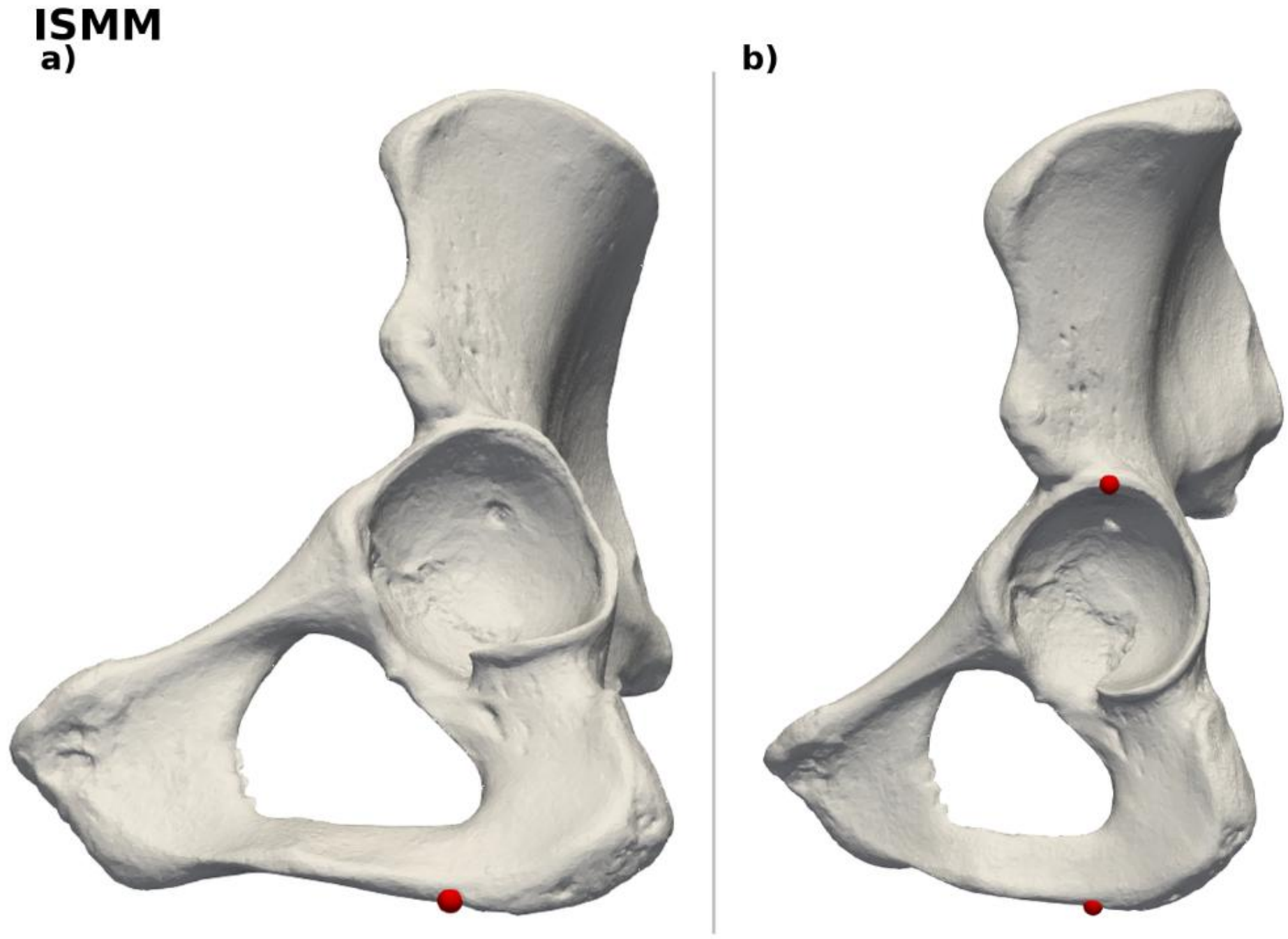

ISMM: (a) Identify the most anteroinferior point of the ischial tuberosity and **(b)** the most distant relevant point on the acetabular rim.

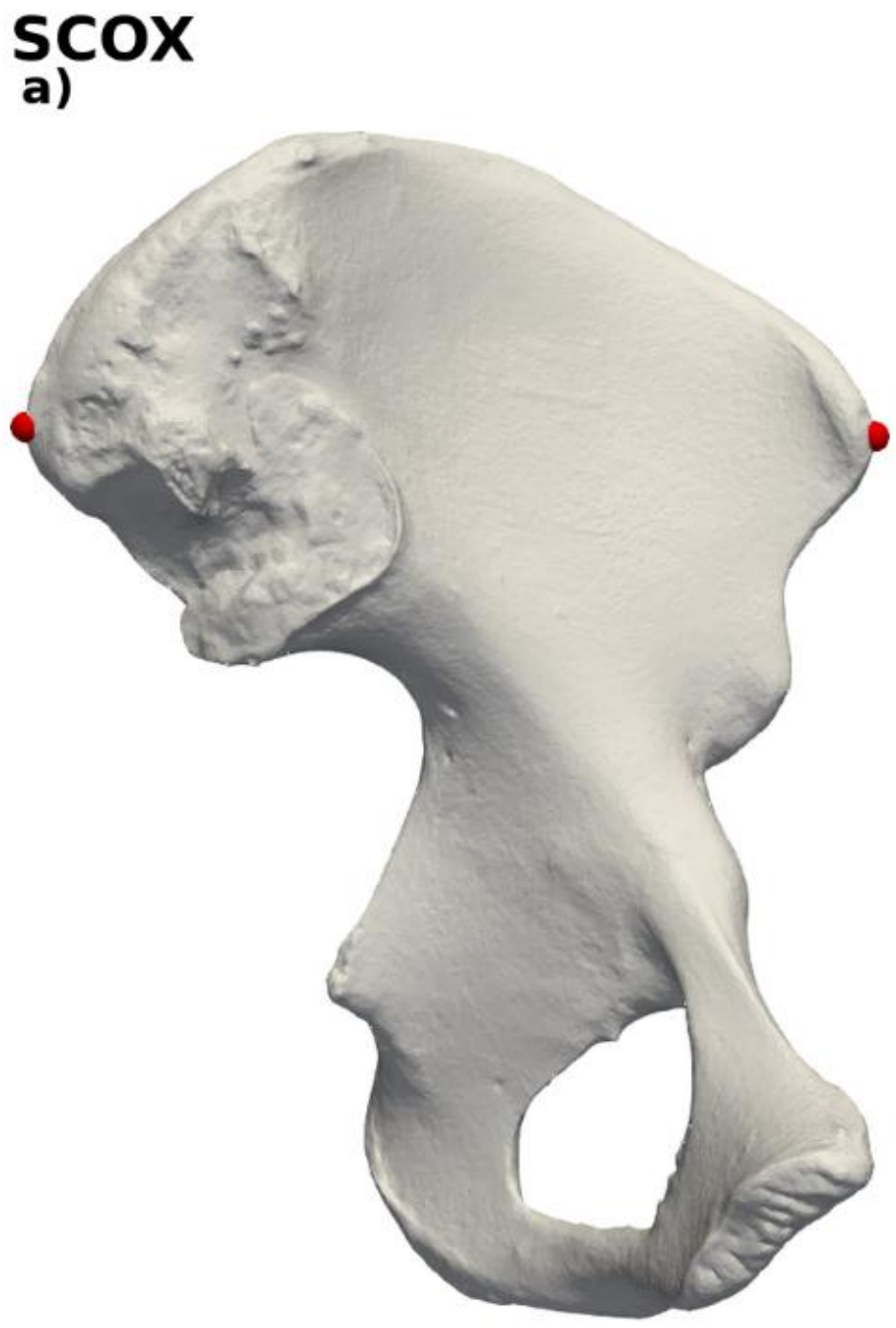

SCOX: (a) Identify the anterior superior iliac spine and posterior superior iliac spine, which define iliac breadth.

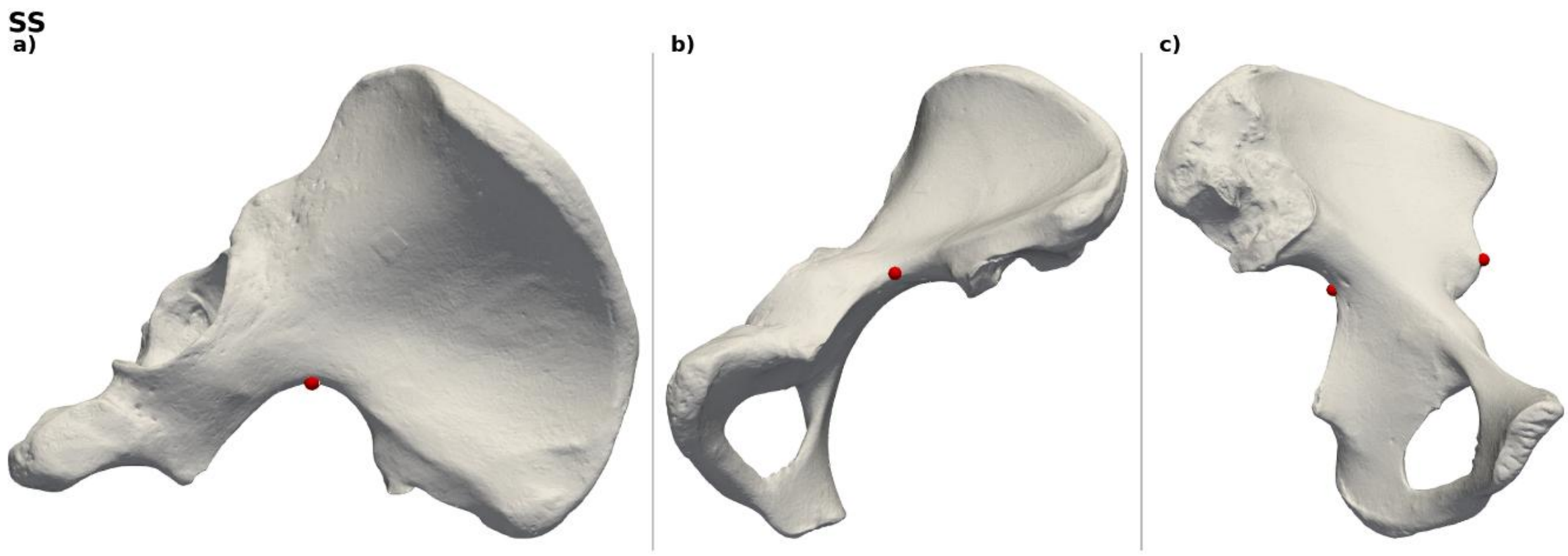

SS: (a) Orient the bone as if placed on a flat surface, then identify **(b)** the deepest point of the greater sciatic notch and (c) the anterior inferior iliac spine.

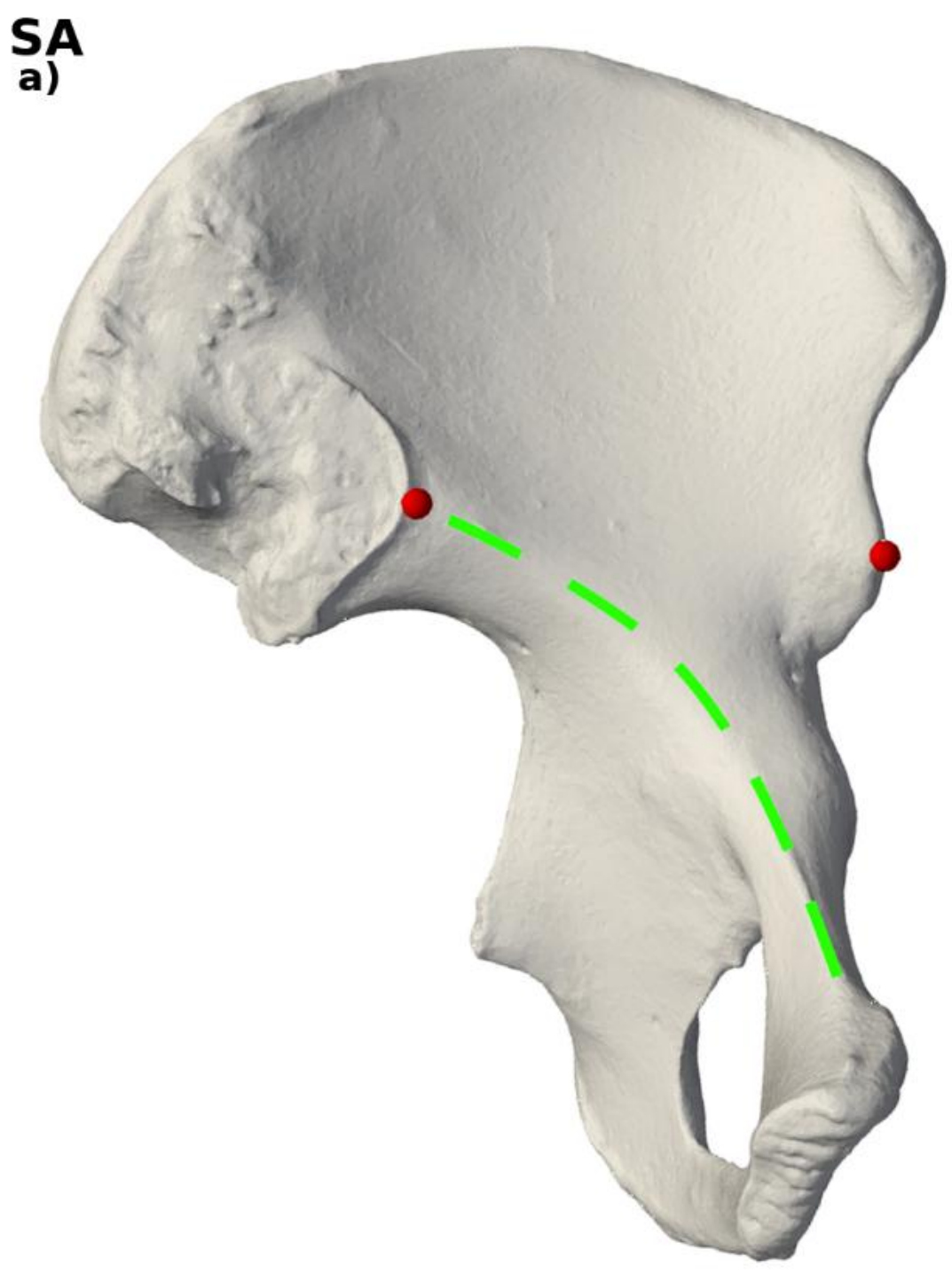

SA: (a) Identify the arcuate line (green) along the superior pubic ramus. The auricular point is then defined as the intersection of the arcuate line with the auricular surface, and SA represents its connection to the anterior inferior iliac spine.

This supplementary guide accompanies the article *From Pocket to Profile: Scale-Free Pelvic Sex Estimation from 3D Photogrammetry* and provides practical instructions for locating the anatomical landmarks and obtaining the seven pelvic measurements used in the proposed workflow. Its purpose is to support consistent application of the method across observers and across both dry-bone specimens and 3D virtual models, particularly in the context of photogrammetric reconstructions.

Full ID of accompanying article:

From Pocket to Profile: Scale-Free Pelvic Sex Estimation from 3D Photogrammetry

International Journal of Legal Medicine

Michal Kuchař1, Alexander Morávek1*, Anežka Pilmann Kotěrová2, Eugénia Cunha3,4, Tomáš Janoušek1

1 Department of Anatomy, Faculty of Medicine in Hradec Králové, Charles University, Šimkova 870, Hradec Králové 500 03, Czech Republic
2 Department of Anthropology and Human Genetics, Faculty of Science, Charles University, Viničná 7, Prague 2 128 44, Czech Republic
3 Department of Life Sciences, Centre for Functional Ecology (CFE), Laboratory of Forensic

Anthropology, University of Coimbra, Calçada Martim de Freitas, Coimbra 3000-456, Portugal
4 Instituto Nacional de Medicina Legal e Ciências Forenses, IP., Lisboa, Portugal

**Corresponding author:*
Alexander Morávek
Department of Anatomy, Faculty of Medicine in Hradec Králové, Charles University, Šimkova 870, 500 03 Hradec Králové, Czech Republic
E-mail: ***moravekal@lfhk.cuni.cz***
